# Oral Tofacitinib and Systemic Corticosteroids, Alone or in Combination, in Patients With Moderate-to-Severe Alopecia Areata: A Retrospective Study

**DOI:** 10.3389/fmed.2022.891434

**Published:** 2022-06-21

**Authors:** Wenxin Zhang, Xiangqian Li, Baifu Chen, Jianzhong Zhang, Kara Melissa T. Torres-Culala, Cheng Zhou

**Affiliations:** ^1^Department of Dermatology, Peking University People’s Hospital, Beijing, China; ^2^Department of Dermatology, Jose R. Reyes Memorial Medical Center, Manila, Philippines

**Keywords:** alopecia areata (AA), Janus kinase inhibitors, tofacitinib, corticosteroids, combination therapy

## Abstract

**Introduction:**

Alopecia areata (AA) is an autoimmune hair loss mediated by CD8 + T cells. Treatment for moderate-to-severe AA is still challenging. Janus kinase inhibitors, such as tofacitinib, have been recently investigated as a promising treatment option for AA. Evidence on the combination use of oral tofacitinib and systemic corticosteroids (SCs) for AA is still lacking.

**Objective:**

To compare the efficacy and safety of monotherapy of oral tofacitinib and SCs, as well as their combination in patients with moderate-to-severe AA.

**Methods:**

Patients with moderate-to-severe AA, who have been treated with at least 3 months of monotherapy of tofacitinib or SCs, or in their combination, were included in this study. The efficacy and adverse events of these treatments were retrospectively analyzed.

**Results:**

Sixty-one patients with moderate-to-severe AA were included in this study. There were 12 (66.7%) of 18 patients in the SCs group, 12 (60.0%) of 20 patients in the tofacitinib group, and 18 (78.3%) of 23 patients achieved SALT_50_, with no significant difference among the three groups. The ratio of patients who achieved SALT_50_ was significantly higher in patients with a short duration of current hair loss episode (≤2 years) than in those with a duration of current hair loss episode (>2 years) in all the three groups. There were 66.7% patients in the SCs group, 35.0% patients in the tofacitinib group, and 56.5% patients in the combined group that showed adverse effects.

**Conclusion:**

Tofacitinib was an effective treatment for patients with moderate-to-severe AA, and it was more tolerated than SCs. A combination of tofacitinib and SCs may have higher efficacy than SCs alone. Efficacy significantly decreased in patients with a current episode of disease for more than 2 years.

## Introduction

Alopecia areata (AA) is an autoimmune non-scarring hair loss, presenting as focal hairless patches to entire scalp loss or loss involving other body hairs (1). The majority of patients with moderate-to-severe AA will experience unpredictable episodes of relapsing and remitting courses or long-term persistence. There is still no reliable therapy for severe AA. The traditional treatments, such as systemic corticosteroids (SCs) and immunosuppressants, are limited in terms of treatment efficacy and high risk of adverse effects (2, 3).

The etiology of AA is related to a complex interaction between genetic and immune abnormalities, which induced inflammation targeting the hair follicles. Breakdown of the immune privilege of hair follicles has been thought to be the prerequisite of AA (4). Interferon-γ (IFN-γ) and CD8 + NKG2D + T cells have been identified as the key contributors to the pathogenesis of AA (5). Evidence from the studies on mouse models of AA has shown that CD8 + NKG2D + T cells release IFN-γ *via* Janus kinase (JAK)1/2 pathways to stimulate interleukin-15 (IL-15) production in follicular epithelial cells. IL-15 then binds to the surface of CD8 + NKG2D + T cells, further promoting the production of IFN-γ *via* JAK1/3 pathways to amplify the inflammatory response and impair the hair growth cycle (6, 7).

Given the important role that JAK pathways play in the pathogenesis of AA, JAK inhibitors (JAKi) have been investigated as a promising therapy for AA. This therapeutic approach could block several signaling pathways and lead to a decrease of CD8 + NKG2D + T cells and a significant improvement of AA. To date, several JAKi have been reported for the treatment of AA, including tofacitinib (JAK1/3), ruxolitinib (JAK1/2), and baricitinib (JAK1/2) (8, 9). Several case reports and studies have demonstrated the impressive efficacy of monotherapy with tofacitinib (10–12), which may expand the landscape of treatment options for moderate-to-severe AA. Interestingly, the combination use of tofacitinib and SCs might induce greater hair growth in several cases resistant to tofacitinib monotherapy (13).

Up to now, evidence on the combination use of oral tofacitinib and SCs for AA is still scarce. Herein, we conducted a retrospective study to compare the efficacy and safety of monotherapy of oral tofacitinib and SCs, as well as their combination in patients with moderate-to-severe AA.

## Methodology

This retrospective study was approved by the Ethics Committee of Peking University People’s Hospital (2021PHB159-001).

The inclusion criteria were Chinese adult patients: (1) diagnosed as AA by two independent dermatologists at the dermatology clinic of the Peking University People’s Hospital between May 2019 and May 2021; (2) with AA subtypes classified as patchy AA, ophiasis, alopecia totalis (AT), and alopecia universalis (AU); (3) with the Severity of Alopecia Tool (SALT) score of 25% or over; and (4) who received at least 3 months of treatment of oral tofacitinib or SCs or combination therapy of the two. Patients with other hair loss disorders, such as androgenetic alopecia, telogen effluvium, trichotillomania, and cicatricial alopecia, were excluded.

The included patients were then divided into the three groups based on the treatment they had received: the SCs group, the tofacitinib group, and the combined therapy group. The demographic and clinical data abstracted from medical records included age, gender, duration of disease, duration of current episode of hair loss, treatment duration, subtypes, and severity of hair loss. Duration of current episode of hair loss was defined as the duration from the latest complete hair regrowth to the time the treatment of SCs and/or tofacitinib was initiated. Laboratory tests, including blood routine test, biochemical blood test, screening for infection of hepatitis B virus, and tuberculosis, were taken before treatment and every 3 months after initiating the treatments.

Alopecia severity was recorded using the SALT score (14). SALT_50_, SALT_90_, and SALT_100_ were defined as 50, 90, and 100% of hair regrowth. The primary endpoint was the ratio of patients who achieved SALT_50_. The secondary outcomes included the ratio of patients who achieved SALT_90_ and SALT_100_, as well as adverse events during the treatments.

Statistical analysis was performed using IBM SPSS software version 26 (IBM SPSS Statistics, Armonk, New York, United States). Data that were normally distributed were expressed as median (range) and non-normally distributed data expressed as median [interquartile range (IQR)]. The one-way ANOVA and the Kruskal–Wallis test were performed as appropriate to compare the continuous variables among the three groups. The chi-squared test and the Kruskal–Wallis test were used as appropriate to compare the non-continuous variables among the three groups. *P*-value < 0.05 was considered as statistically significant.

## Results

### Patient Characteristics

This study involved 61 patients (38 women and 23 men) with a median age of 29.7 years. There were 18, 20, and 23 patients in the SCs group, the tofacitinib group, and the combined group, respectively. Patients’ demographic and clinical characteristics of the three groups are given in Table 1.

**TABLE 1:** Demographic data and characteristics of patients with AA in each group.

	SCs group (*n* = 18)	Tofacitinib group (*n* = 20)	Combined group (*n* = 23)	*P*-value
Age, median (IQR), year	29.4 (19.9–44.8)	26.1 (22.8–32.6)	35.9 (28.8–43.4)	0.060
Sex, n (%)				0.781
Male	8 (44.4%)	7 (35%)	8 (34.8%)	
Female	10 (55.6%)	13 (65%)	15 (65.2%)	
Severity, n (%)				0.351
Moderate AA	8 (44.4%)	8 (40.0%)	7 (30.4%)	
Severe AA	10 (55.6%)	12 (60.0%)	16 (69.6%)	
Duration of disease, median (IQR), year	1.8 (0.3–14.4)	9.7 (4.5–15.4)	5.2 (1.0–7.8)	0.046
Duration of current disease episode, median (IQR), year	1.1 (0.3–7.7)	4.0 (0.8–9.6)	2.2 (0.8–6.5)	0.364
Treatment duration, median (IQR), months	7.1 (4.2–14.4)	12.1 (7.0–16.5)	9.3 (5.3–12.9)	0.334
Alopecia areata subtypes, n (%)				0.607
Patchy AA	8 (44.4%)	10 (50%)	11 (47.8%)	
Ophiasis	2 (11.1%)	4 (20%)	5 (21.7%)	
Alopecia totalis	1 (5.6%)	3 (15%)	1 (4.3%)	
Alopecia universalis	7 (38.9%)	3 (15%)	6 (26.1%)	
Initial SALT score, median (IQR),%	55.0 (34.1–95.0)	50.0 (32.5–87.5)	60.0 (40.0–80.0)	0.913
SALT change, median (range),%	97.5 (4.76–100.0)	85.6 (0–100.0)	92.0 (50.0–100.0)	0.399
Continuing treatment at study conclusion, n (%)	1 (5.6%)	11 (55%)	14 (60.9%)	

*SCs, systemic corticosteroids; IQR, interquartile; AA, alopecia areata; SALT, Severity of Alopecia Tool.*

For the combined group, 60.9% (14/23) of patients were treated initially with tofacitinib and SCs in combination. However, 26.1% (6/23) of patients initiated with SCs alone and 13.0% (3/23) of patients initiated with tofacitinib alone did not show favorable response, and they subsequently tried combination therapy. For the dosage, 56.5% (13/23) and 43.4% (10/23) of patients in the combined group were treated initially with 5 mg tofacitinib two times daily or 5 mg one time daily. All the patients in the tofacitinib group were treated with 5 mg two times daily initially. Based on the treatment responses, the dose of tofacitinib in the two groups was then adjusted to 5 or 15 mg daily. The ways of administrating SCs included: (1) intramuscular betamethasone pulse therapy for 3–4 months, followed by tapering oral prednisone, (2) oral daily prednisone from the beginning at a dosage of 15–30 mg (tapering when observed fully hair regrowth without any dermoscopic sign of disease activity), and (3) oral prednisone daily at a dosage of 15–30 mg combined with intramuscular betamethasone at a dosage of 0.3–1 ml monthly. These ways of SC administration were done for both the SCs group and the combined group. A total of 1 ml betamethasone was estimated as equivalent to a dose of 70 mg prednisone.

### Efficacy

**FIGURE 1:**
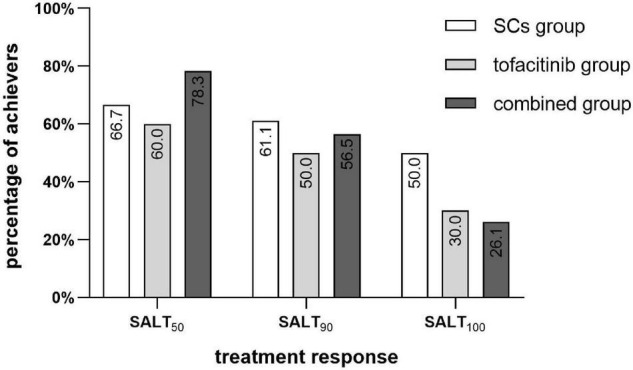
Percentage of SALT_50_, SALT_90_, and SALT_100_ achievers in the SCs group (*n* = 18), the tofacitinib group (*n* = 20), and the combined group (*n* = 23). SCs, systemic corticosteroids; SALT, Severity of Alopecia Tool.

**FIGURE 2:**
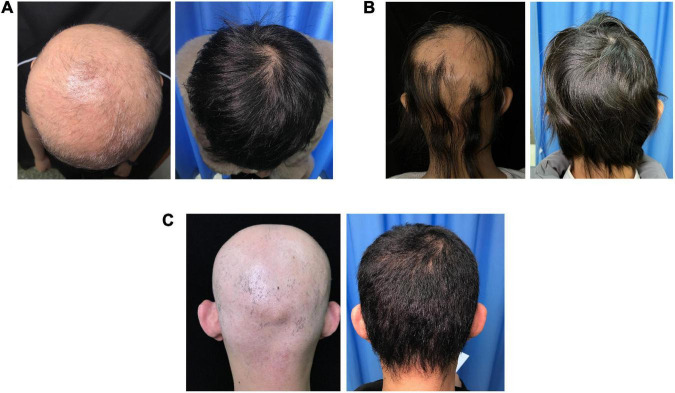
Photographs of three representative patients in the three groups. **(A)** Patient in the SCs group with the SALT score of 100% prior to treatment and 0% after 18 months of treatment. **(B)** Patient in the tofacitinib group with the SALT score of 75% prior to treatment and 0% after 7 months of treatment. **(C)** Patient in the combined group with the SALT score of 100% prior to treatment and 0% after 31 months of treatment.

Treatment responses in each group are shown in Figure 1. The percentage of patients who achieved SALT_50_, SALT_90_, and SALT_100_ was not significantly different among the three groups (*P* = 0.423, 0.785, and 0.245, respectively). Photos of representative responders in the three groups are shown in Figure 2.

In the SCs group, 66.7% (12/18) of patients achieved SALT_50_. The median treatment duration was 6.2 months (range 3.3–15.2 months) and the median total prednisone equivalent dose was 1,105 mg (range 280–4,780 mg). 61.1% (11/18) of patients achieved SALT_90_ (median 5.3 months of treatment) and 50.0% of patients achieved SALT_100_ (median 5.1 months of treatment).

In the tofacitinib group, 60.0% (12/20) of patients achieved SALT_50_. The median treatment duration was 12.5 months (range 3.3–20.7 months) and the median total prednisone equivalent dose was 3,150 mg (range 900–7,800 mg). 50.0% (10/20) of patients achieved SALT_90_ (median 13.2 months of treatment) and 30.0% of patients achieved SALT_100_ (median 14.4 months of treatment).

In the combined group, 78.3% (18/23) of patients achieved SALT_50_. The median treatment duration was 10.4 months (range 4.1–39.0 months) with a median total prednisone equivalent dose of 880 mg (range 154–3,300 mg) and median total tofacitinib dose of 2,100 mg (range 600–11,400 mg). 56.5% of patients achieved SALT_90_ (median 8.0 months of treatment) and 26.1% of patients achieved SALT_100_ (median 6.4 months of treatment).

In the SCs group, the ratio of SALT_50_ achievers was significantly higher in patients with a short duration of current hair loss episode (≤2 years) than in those with a duration of current hair loss episode (>2 years) (90.9 vs. 28.6%, *P* = 0.006). Similarly, the ratio of SALT_50_ achievers was significantly higher in patients with a shorter duration of current hair loss episode in the tofacitinib group (100.0 in ≤ 2 years subgroup vs. 33.3% in > 2 years subgroup, *P* = 0.003) and in the combined group (100.0 vs. 58.3%, *P* = 0.016).

In addition, for patients who had more than 10 years of current episode of scalp hair loss, there were 0 (0/4), 75.0 (3/4), and 66.7% (2/3) of patients in the SCs group, the tofacitinib group, and the combined group that achieved SALT_50_, respectively.

**FIGURE 3:**
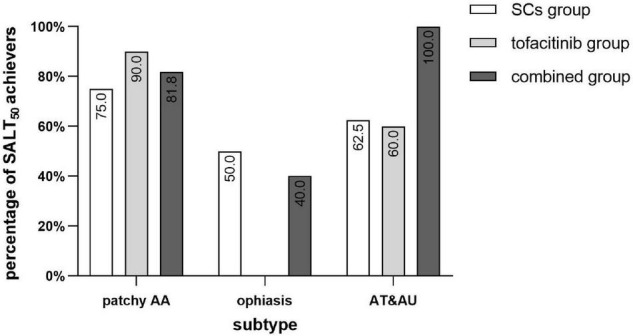
Percentage of SALT_50_ achievers of different subtypes of AA in the three groups. SCs, systemic corticosteroids; SALT, Severity of Alopecia Tool; AA, alopecia areata; AT, alopecia totalis; AU, alopecia universalis.

For patients with AT and AU, there were 62.5 (5/8), 60.0 (3/5), and 100% (7/7) of patients in the SCs group, the tofacitinib group, and the combined group that achieved SALT_50_, respectively (Figure 3). For patients with ophiasis, there were 50.0 (1/2), 0 (0/4), and 40.0% (2/5) of patients in the SCs group, the tofacitinib group, and the combined group that achieved SALT_50_, respectively.

In the combined group, 80.0% (4/5) of patients who achieved SALT_50_ and discontinued the treatment experienced recurrence after a mean duration of 3.3 months. In the tofacitinib group, 66.7% (2/3) of patients who achieved SALT_50_ had completely stopped the treatment and recurred after a mean duration of 2 months. In the SCs group, 60.0% (6/10) of patients who achieved SALT_50_ showed recurrence after stopping the treatment in a mean duration of 2 months.

### Safety

The adverse effects (AEs) are given in Table 2. All of the AEs were mild in the three groups and none of the patients discontinued the treatments because of the AEs. The proportions of patients who experienced AEs were not significantly different between the three groups (*P* = 0.132).

**TABLE 2:** AEs observed in each group*.

	SCs group (*n* = 18)	Tofacitinib group (*n* = 20)^a, c^	Combined group (*n* = 23)^c^
Patients who experienced AEs, n(%)	12 (66.7%)	7 (35.0%)	13 (56.5%)
Infections (n)	Acne (3)	Acneiform eruption (4)^b^	Folliculitis (4)
		Zoster (1)	Acneiform eruption (6)^b^
			Wart (1)
			Upper respiratory infection (2)
Other AEs (n)	Weight gain (8)	Fatigue (1)	Liver enzyme abnormalities (2)
	Moon facies (2)	Hyperlipidemia (2)	Hyperlipidemia (2)
	Arthralgia (1)	Arthralgia (1)	Hirsutism (3)
	Hirsutism (1)		Weight gain (5)
	Headache (1)		Proteinuria (1)
	Menstrual irregularity (1)		Fatigue (1)
	Hypertension (1)		Moon facies (1)
	Sleep disturbance (1)		Menstrual irregularity (1)
	Frequent urination (1)		

*The proportions of patients who experienced AEs were not significantly different between the three groups (P = 0.132).

*^a^One patient was diagnosed with asymptomatic liver enzyme elevation before treatment, which didn’t increase during the therapy.*

*^b^Patients who experienced acneiform eruption mostly performed as whitehead.*

*^c^Two patients in the combined group and the tofacitinib group were diagnosed with hepatitis before the treatment. Both of them experienced elevated HBV DNA during the treatment without any liver enzyme abnormalities and liver symptoms, one of whom is taking anti-virus medication for now.*

*SCs, systemic corticosteroids; AEs, adverse effects.*

In the SCs group, 66.7% (12/18) of patients experienced AEs, including weight gain (8), acne (3), moon facies (2), hirsutism (1), arthralgia (1), headache (1), menstrual irregularity (1), hypertension (1), sleep disturbance (1), and frequent urination (1).

In the tofacitinib group, 35.0% (7/20) of patients showed AEs, including acneiform eruption (4), recurrent herpes zoster (1), fatigue (1), hyperlipidemia (2), and arthralgia (1). One patient in the tofacitinib group was diagnosed with asymptomatic liver enzyme elevation before treatment, which did not increase during the therapy with tofacitinib.

In the combined group, 56.5% (13/23) of patients showed AEs. Folliculitis (4) and acneiform eruption (6) were the most common infectious AEs, followed by upper respiratory infection (URI) (2) and wart (1). Weight gain (5), hirsutism (3), hyperlipidemia (2), liver enzyme abnormalities (2), proteinuria (1), fatigue (1), and moon facies (1) were also reported in the combined group of patients. It is worth mentioning that two patients in the combined group and the tofacitinib group had been diagnosed with hepatitis B before treatment, and after being refused tofacitinib by our dermatologists, they started the treatment by themselves with illegally imported tofacitinib for 39 and 16.6 months. One of the two patients had been taking antiviral medication and both of them experienced elevated hepatitis B virus (HBV) DNA levels during the treatment, without liver enzyme abnormalities and liver symptoms.

## Discussion

To date, despite the numerous options that have been proposed for systemic treatment of AA, the majority of them are of limited efficacy with a high risk of side effects and high recurrence rates, especially for patients with severe AA. Previous studies have shown favorable results of SCs in patients with AA, but a lower response rate in ophiasis and AU (15, 16). Additionally, the response may not be maintained and the majority of patients will relapse within 4–9 weeks after discontinuing SCs treatment (2), which is consistent with our findings.

Some studies and case series have demonstrated promising efficacy of tofacitinib as a monotherapy of 10 mg/day or higher doses in adult patients with severe AA (11, 17, 18). In a clinical trial of 66 adults with severe AA treated with 10 mg of tofacitinib daily, it was shown that SALT_50_ was achieved in 32.0% of patients after 3 months (11). In a retrospective study of 18 patients with refractory AT or AU, 44.4% of patients achieved SALT_50_ with a 10–15 mg daily dose of tofacitinib after 6 months (17). In this study, patients in the tofacitinib group presented slightly higher efficacy (60.0% of patients achieved SALT_50_). It was probably due to that, moderate AA was included and treatment duration was longer in this study.

Combined treatment showed probably a higher response rate in SALT_50_ than monotherapy of tofacitinib or SCs; however, they did not achieve any statistical significance. Studies with a larger sample size are needed to clarify this. Interestingly, our limited data did not show higher SALT_90_ and SALT_100_ in combined treatment than in monotherapy.

Some previous reports indicated that patients resistant to SCs may respond to tofacitinib (17, 19, 20). Meanwhile, it has been reported that some patients that resistant to tofacitinib monotherapy could respond to combination therapy of tofacitinib and prednisone (13). Notably, in the combined group of this study, 26.1% (6/23) of patients and 13.0% (3/23) of patients who did not show significant hair regrowth when receiving monotherapy of SCs or tofacitinib, respectively, achieved SALT_50_ after receiving combined therapy. This implicated that the immunological pathogenesis of AA is still far from clear.

It is worth noting that 100% of patients with AT and AU achieved SALT_50_ in the combined group, while only about 60% of patients with AT and AU achieved SALT_50_ in the SCs or tofacitinib monotherapy group. It was considered that the larger the area of hair loss, the poorer the treatment response, especially in AT and AU (21). In patients with any monotherapy, patchy AA responded better than AT and AU; however, in the combined group, patients with AT and AU seemed to show a better treatment response than patchy AA. Ophiasis is a special subtype of AA associated with poor prognosis and reduced efficacy to traditional systemic treatment (2). In a previous clinical trial, three patients with ophiasis who were treated with tofacitinib for 3 months showed a median SALT improvement of 68% and were more responsive than patients with AT and AU (11). For ophiasis in this study, though 50% in the SCs group and 40% in the combined group achieved SALT_50_, no patients in the tofacitinib group showed favorable efficacy. Interestingly, patients with AT and AU showed more favorable responses than patients with ophiasis in all the three different treatment groups.

Duration of current episode of hair loss has been recognized as a probable clinical indicator to predict treatment response in some previous reports (12). In this study, we found that the efficacy of patients with AA who had more than 2 years of current episode of disease in all the three groups was significantly poorer compared with patients who had less than 2 years of current episode of disease. Interestingly, for patients with more than 10 years of duration of current episode of disease, there was no patient in the SCs group who achieved SALT_50_, while there were 75.0 and 66.7% of patients in the tofacitinib group and the combined group who achieved SALT_50_, respectively. It seemed that tofacitinib with or without SCs might be a better choice than SCs alone for moderate or severe patients with AA with long disease duration.

Relapse occurred after cessation of therapy in monotherapy of tofacitinib or SCs or their combination, which indicated that maintenance therapy might be necessary for obtaining continued remission.

Though different application strategies of SCs have shown fair efficacy in severe AA, high rates of AEs have restricted the long-term application of SCs (2, 3). In a placebo-controlled trial, 23 patients with severe AA were treated with weekly oral prednisolone pulse therapy (200 mg). AEs were seen in 55.0% of patients after 3 months of treatment and 3 months of follow-up, including general weakness, acneiform eruption, weight gain, facial mooning, and gastrointestinal upset (22). In this study, though no serious AEs were recorded, patients in the SCs monotherapy group also showed the highest rate of AEs (66.7%) among the three groups.

The AEs occurred in patients with tofacitinib monotherapy seemed to be mild, self-limited, and more acceptable for patients than SCs. Severe AEs reported in tofacitinib treatment, including severe infections, venous thromboembolic events, and malignancy, were not reported in our study. The most frequently reported AE of tofacitinib is the increased risk of infection (23), such as nasopharyngitis and URI (24). Interestingly, in this study, only two patient in the combined group experienced URI, while none in the monotherapy group with SCs or tofacitinib experienced URI. One possible reason for the low incidence of URI might be the increasing use of facial masks for preventing COVID-19 in China since January 2020. In our experience, patients who are undergoing treatments of immunosuppressant and/or systemic corticosteroids should be encouraged to wear a face mask in assembly occupancies for preventing URI. The incidence of acneiform eruption in the tofacitinib group and the combined group was remarkably higher than it was reported in previous studies. In a retrospective study, no HBV reactivation was observed in four patients with rheumatoid arthritis with resolved HBV infection treated with tofacitinib within 3 years of follow-up (25). It is worth noting that two patients with hepatitis B infection were included in the combined group and the tofacitinib group, one of whom accepted antiviral medication. Both of them experienced elevated HBV DNA levels during the treatment, without liver enzyme abnormalities and liver symptoms. Tofacitinib appears safe in patients with resolved HBV infection (26) and reactivation of HBV infection could be prevented by antiviral prophylaxis. However, it is important to beware of the high incidence rate of HBV reactivation in patients with HBsAg + receiving tofacitinib and close monitoring of HBV DNA and alanine aminotransferase should be suggested (27). In clinical trials of tofacitinib for rheumatoid arthritis, no new AEs were observed in 10 years (28). However, concerns still exist regarding its long-term safety profile, and longer observational periods are still needed.

Tofacitinib is a non-specific JAK inhibitor, and it can target more than one single JAK molecule and may lead to a relatively broad immune suppression (29). More selective JAKi are expected to increase efficacy and improve safety in the future. Topical JAKi are currently under investigation in patients with AA for the sake of minimizing the risk of systemic side effects, especially for the long-term treatment (30). However, the efficacy of topical JAKi has not yet been shown as encouraging as oral tofacitinib for severe AA (24).

In summary, tofacitinib was an effective treatment for patients with moderate-to-severe AA and it was more tolerated than corticosteroids. Oral tofacitinib combined with SCs seems to have higher efficacy with lower dosage and less AEs than SCs alone. Efficacy decreased in patients with current episode of disease for more than 2 years, regardless of treatments with oral tofacitinib, SCs, or their combination.

## Limitation

An intrinsic limitation of this study is that it is a retrospective study. Though we had tried to minimize the differences in baseline characteristics of the groups, there was still difference in duration of disease among the three groups, which had the potential to impact the results biaswise. The SALT scores in some follow-ups during the treatment were missing; therefore, the association of the SALT score changes and treatment duration were not evaluated in this study. The sample size of this study is relatively small, and significant difference in the efficacy was not identified among the three treatment groups. Studies with larger sample sizes will still be needed in the future.

## Data Availability Statement

The raw data supporting the conclusions of this article will be made available by the authors, without undue reservation.

## Author Contributions

CZ and WZ contributed to the conception and design of this study, collected the data, and wrote and edited the manuscript. WZ and XL performed the statistical analysis. CZ reviewed the literature. KT-C edited the manuscript. All authors have contributed to the article and approved the submitted version of the manuscript.

## Conflict of Interest

The authors declare that the research was conducted in the absence of any commercial or financial relationships that could be construed as a potential conflict of interest.

## Publisher’s Note

All claims expressed in this article are solely those of the authors and do not necessarily represent those of their affiliated organizations, or those of the publisher, the editors and the reviewers. Any product that may be evaluated in this article, or claim that may be made by its manufacturer, is not guaranteed or endorsed by the publisher.
